# Risk of Shiga Toxigenic* Escherichia coli* O157:H7 Infection from Raw and Fermented Milk in Sokoto Metropolis, Nigeria

**DOI:** 10.1155/2018/8938597

**Published:** 2018-05-15

**Authors:** Yusuf Yakubu, Abdulmalik Bello Shuaibu, Aliyu Musawa Ibrahim, Ummukulthum Lawal Hassan, Raymond Junior Nwachukwu

**Affiliations:** ^1^Department of Veterinary Public Health and Preventive Medicine, Faculty of Veterinary Medicine, Usmanu Danfodiyo University, Sokoto, Nigeria; ^2^Department of Veterinary Microbiology, Faculty of Veterinary Medicine, Usmanu Danfodiyo University, Sokoto, Nigeria; ^3^College of Agriculture and Animal Science, Bakura, Zamfara State, Nigeria

## Abstract

*Escherichia coli* O157:H7 is an enteric foodborne pathogen associated with life threatening disease conditions. The enterobacteria are frequently found in cattle gastrointestinal tract with high potential of contaminating animal products such as meat, milk, and cheese. A cross-sectional study was conducted to investigate the presence of Shiga toxin-producing* Escherichia coli* O157:H7 in milk products sold within Sokoto metropolis. Two hundred and sixty (260) samples (comprising 160 raw and 100 fermented milk samples) were collected from different sources within the study area. Bacteriological isolation and biochemical characterization yielded* Escherichia coli* with a detection rate of 9.23% (24/260). Molecular identification of the recovered isolates by PCR amplification of the* Stx1* gene revealed* Escherichia coli* O157:H7 with a positive rate of 20.83% (5/24). The overall prevalence of* E. coli* O157:H7 was 1.92% (5/260) and the positive proportions for raw and fermented milk samples were 1.86% (3/160) and 2.0% (2/100), respectively. Fisher's Exact test showed a nonsignificant association between the isolates and the different milk types (*p* = 0.943; OR = 0.94; [95% CI: 0.154–5.704]). The results revealed presence of* Escherichia coli* O157:H7 in raw and fermented milk sold within Sokoto metropolis, Nigeria. The findings indicate possible feacal contamination of the milk products, with serious public health consequences. This necessitates the need to screen other milk products produced in the area such as butter and cheese. Health authorities in the State need to enlighten dairy farmers on the zoonotic potential of* Escherichia coli* O157:H7 and the role of cattle in the spread of the pathogen.

## 1. Introduction

*Escherichia coli* is a Gram-negative, rod shaped, facultative anaerobic bacterium of the family Enterobacteriaceae. Based on its virulence, the bacterial organism is classified into five groups, namely, enterotoxigenic* E. coli* (ETEC), enteropathogenic* E. coli* (EPEC), enterohemorrhagic* E. coli* (EHEC), attaching and effacing* E. coli* (AEEC), and Shiga toxin-producing* E. coli* (STEC) [1].* Escherichia coli* O157:H7 is an emerging serotype of* Escherichia coli *that accounts for most human diseases caused by enterohaemorrhagic* Escherichia coli *(EHEC). It is associated with life threatening disease conditions in humans such as hemolytic uremic syndrome (HUS), thrombotic thrombocytopenic purpura (TTP), and hemorrhagic colitis (HC) [2]. The organism was first recognized as a human pathogen following outbreaks of hemorrhagic colitis associated with consumption of contaminated beef hamburger [3]. Cattle are the principal reservoir of* Escherichia coli* O157:H7 and are resistant to infection by the pathogen as they lack receptors responsible for the attachment of the bacteria to host cell [4]. However, humans are highly susceptible to infection following direct contact with contaminated animal faeces or consumption of contaminated animal products such as beef, milk, and cheese [5, 6].* Escherichia coli* O157:H7 has worldwide distribution and determinants responsible for its emergence in human population are yet to be confirmed [7, 8]. However, changes in livestock husbandry system, slaughter, and meat processing practices have been suggested to play important role in the emergence of the pathogen [9]. Enterohemorrhagic* Escherichia coli* expresses one or more potent cytotoxins known as Shiga toxins (Stx), which are the major virulence factors in the pathogenesis of diseases caused by the bacteria.* Escherichia coli* O157:H7 produces Shiga toxin 1 which is encoded by the* Stx1* gene and serologically indistinguishable with the toxin of* Shigella dysenteriae* [10].

Cow milk forms an important component of some traditional dairy dishes and beverages of the Fulani and Hausa tribes in northern Nigeria. It is sometimes served raw immediately after milking or allowed to ferment for use as a major recipe in a popular staple beverage known as “*fura da nono.*” There is growing health concern over the unhygienic manner of hand milking by some livestock farmers in the study area. Similarly, the habitual consumption of unpasteurized milk and its products presents considerable risk of milk-borne infections to consumers. This study was aimed at investigating the presence of Shiga toxin-producing* E. coli* O157:H7 in raw and fermented milk sold within Sokoto metropolis using culture and molecular methods.

## 2. Materials and Methods

### 2.1. Study Area

The study area was Sokoto metropolis, the capital of Sokoto State Nigeria. It is made up of four local government areas, namely, Sokoto North, Sokoto South, Wamakko, and Dange-Shuni. The State is located on latitude 13°N and between longitudes 4°8′E and 6°54′E in Northwestern Nigeria. The State covers an area of approximately 56,000 square kilometers [11]. The State shares border with Niger republic to the north, Kebbi State to the south, and Zamfara State to the east. Based on the 2006 census, Sokoto State was estimated to have a population of about 4,344,399. The State is ranked second in livestock population with about 3 million cattle, 4 million goats, 3.85 million sheep, 0.8 million camels, and 1 million poultry [12].

### 2.2. Study Design and Sample Collection

A cross-sectional study was conducted where dairy cattle herds and milk retailing outlets within Sokoto metropolis were identified for the collection of raw and fermented milk, respectively. A total of 160 raw milk samples were collected from 16 dairy herds within 3 Local Government Areas (LGAs) comprising 62 samples from Sokoto North, 55 samples from Sokoto South, and 43 samples from Dange-Shuni. Sampling could not be done in Wamakko LGA as there are no established dairy cattle herds. To collects samples, lactating cows were randomly selected and 10 ml of milk was collected in sterile bottles from each cow by the livestock attendants. Ten milliliters (10 ml) of pooled fermented milk samples was also collected from 100 different sales outlets within the metropolis. Both milk samples (raw and fermented) were transported in an ice chest to the Public Health Laboratory, Faculty of Veterinary Medicine, Usmanu Danfodiyo University Sokoto, Nigeria.

### 2.3. Culture and Biochemical Characterization

The acidity of fermented milk samples was ascertained using a pH indicator paper (Whatman®). Both the raw and fermented milk samples were diluted tenfolds using sterile distilled water before inoculating 1 ml onto MacConkey agar (Oxoid, UK) and subcultured on Eosin Methylene Blue agar (Oxoid, UK) using spread plating technique. The plates were incubated at 37°C for 24 hours and bacterial colonies that are circular, moist, smooth, and pinkish on MacConkey agar but have green metallic sheen on Eosin Methylene Blue agar were presumed to be* Escherichia coli.* All presumptive colonies were subjected to a panel of conventional biochemical tests (IMViC) and isolates that were positive for indole and methyl red tests but negative for Voges Proskauer and Citrate utilization tests we identified as* E. coli*. The identified* E. coli* isolates were further subcultured onto sorbitol MacConkey agar (Oxoid, UK) and incubated at 37°C overnight. Nonsorbitol fermenters that appear as smooth, circular, and colourless colonies were tentatively identified as* Escherichia coli *O157:H7 as earlier described [13].

### 2.4. DNA Extraction

The boiling method of nucleic acid extraction was employed as earlier described [14]. Briefly, a loopful of each isolate was suspended in two hundred microliters (200 *μ*l) of TE buffer (Tris-HCl [10 mM]: EDTA [1 mM]) in a microfuge tube and heated at 96°C for 15 mins. The tubes were immediately transferred onto ice for 15 mins and then centrifuged at 15000 g for 2 mins at room temperature. The pellets were discarded while the supernatant containing the DNA templates was used for polymerase chain reaction.

### 2.5. Molecular Identification

A multiplex polymerase chain reaction (PCR) was done using TopTaq™ Master Mix PCR kit (Qiagen®). The 25 *μ*l reaction mixture contained 12.5 *μ*l TopTaq Master Mix 2x, 7.5 *μ*l RNase-free water, 2.5 *μ*l of 200 ng DNA template, and 0.25 *μ*M of two-primer cocktail (Table 1) amplifying the 180 bp of the* Stx1* gene as described earlier [15]. Samples were subjected to 35 PCR cycles, each consisting of 1 min of denaturation at 95°C, 2 min of annealing at 65°C for the first 10 cycles, decrementing to 60°C by cycle 15, and 1.5 min of elongation at 72°C, incrementing to 2.5 min from cycles 25 to 35. Sterile RNase-free water and a confirmed* E. coli* O157:H7 isolate were used as negative and positive controls, respectively.

### 2.6. Agarose Gel Electrophoresis

A 1.5% agarose gel was prepared by suspending 1.5 grams of agarose powder in 100 ml of 1x Tris-Borate-EDTA (TBE) buffer and heated on a hot plate until completely dissolved. Ethidium bromide (2.5 *μ*l) was added to the liquid agarose before pouring into a gel caster set and allowed to solidify at room temperature. The caster was rightly placed into an electrophoresis tank and flooded with 1x TBE buffer to the maximum level before carefully removing the comb. The PCR products (5 *μ*l each) were then loaded into the wells using a 10 *μ*l Eppendorf pipette. Four microliters (4 *μ*l) of 100 bp ladder (BioLabs Inc. New England) mixed with 2 *μ*l Gel Loading Dye Blue 6x (BioLabs Inc. New England) was also loaded into one of the wells before connecting the tank to a power pack and plugging it to the mains supply. The products were electrophoresed at 85 volts for 50 mins. Immediately after electrophoresis, the agarose gel was viewed using a Gel Doc™ XR+ (BioRad). The gel image was captured and labelled accordingly.

### 2.7. Data Analyses

The results obtained were presented in tables and narratives. Fisher's Exact test was used to determine association between the* E. coli* O157:H7 isolates and type of milk (raw and fermented) using a significance level of 5% and 95% confidence interval. All analyses were performed using SPSS (version 23; SPSS Inc., Chicago, IL, USA).

## 3. Results and Discussion

**Figure 1:**
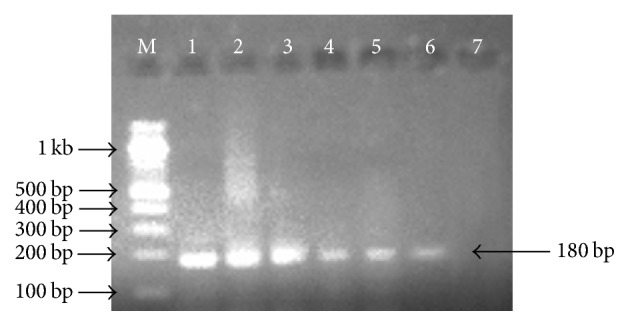
Agarose gel electrophoresis result for PCR detection of* Stx1* gene in* Escherichia coli* O157:H7. Lane M: 100 bp ladder (BioLabs), lane 1: positive control, lanes 2 to 6: positive samples, and lane 7: negative control.

The overall prevalence of* E. coli* was 9.23% (24/260) with an isolation rate of 9.38% (15/160) and 9.0% (9/100) for raw and fermented milk, respectively. Statistical analysis using Chi Square (*χ*^2^) test showed a nonsignificant association (*p* = 0.919, OR = 1.05, [95% CI: 0.44–2.49]) between the* E. coli* isolates and the different milk types (Table 2). Molecular identification by PCR amplification of the Shiga toxin 1 gene* (Stx1)* in* E. coli* O157:H7 showed 5 out of the 24* E. coli* isolates to be positive with a prevalence of 20.83% (5/24) (Figure 1). The prevalence in raw and fermented milk was 1.86% (3/160) and 2.0% (2/100), respectively. Fisher's Exact test revealed a nonsignificant association (*p* = 0.943; OR = 0.94; [95% CI: 0.154–5.704]) between the* E. coli* O157:H7 isolates and the different milk types (Table 3). The proportions of raw milk samples positive for* Escherichia coli* in three local government areas sampled were 5.45% (3/55), 12.70% (8/62), and 9.30% (4/43) for Sokoto South, Sokoto North, and Dange-Shuni, respectively. Univariable analysis by logistic regression showed a nonsignificant association between the* E. coli* isolates and the local government areas (Table 4). However, only 3 isolates of* E. coli *recovered from raw milk samples were* E. coli *O157:H7 with positive proportions of 25.0% (2/8) and 33.3% (1/3) for Sokoto North and Sokoto South LGAs, respectively. None of the* E. coli* isolates from Dange-Shuni LGA were STEC. Fisher's Exact test revealed a nonsignificant association (*p* = 0.998; OR = 1.50; [95% CI: 0.084–26.86]) between the STEC isolates and the LGAs in the study area (Table 5). The two STEC identified among the nine* E. coli* isolated recovered from fermented milk were from different LGAs (Sokoto North and Sokoto South). However, no statistical analysis was undertaken to determine any association between the STEC isolates and the LGAs since both areas had one positive sample each.

Milk is a good medium for the growth and proliferation of microorganisms and several disease causing bacteria such as* Listeria monocytogenes*,* Salmonella* species,* Shigella* species, and* Campylobacter* species have been associated with consumption of contaminated milk [16]. Of all the pathogens responsible for milk-borne diseases,* E. coli* O157:H7 is the most pathogenic as it has been incriminated in various disease conditions such as hemolytic uremic syndrome, thrombotic thrombocytopenic purpura, and hemorrhagic colitis [2]. Moreover, studies on transmission dynamics of the pathogen have reported similar virulence profiles in isolates recovered from humans and food-producing animals such as cattle [16]. The isolation of* E. coli* O157:H7 from both raw and fermented cow milk in this study indicates potential fecal shedding of the pathogen and subsequent contamination of the milk. Cattle are the major reservoirs of the organism and can actively shed the bacteria in faeces without any clinical manifestation [5, 17]. Thus there is high risk of milk contamination following unhygienic collection from infected animals or herds with carriers of the pathogen as reported [18]. Hand milking is the most practiced method of milk collection among the Hausa/Fulani nomadic herds in Nigeria. These livestock farmers own over 90% of the cattle population in the country and do not practice premilking hygiene such as washing of hands and cleaning/disinfection of udder teats [19]. Moreover, they do not pasteurize milk as they are not properly educated on the health risk of consuming contaminated milk [20]. Therefore, milk supplied by these dairy farmers is potentially contaminated with pathogenic flora from the udder or teat canal and the animal's skin, thus posing serious health threat to consumers.

Fermentation of milk is used as a means of preservation by lowering the pH with resultant reduction in microbial load. It is one of the oldest means of milk preservation where unpasteurized milk is left to undergo spontaneous fermentation by microorganism present in the milk and the surrounding air. In most traditions, inoculum of unknown microbial identity from previously fermented milk is used as starter culture. However, this practice reintroduces potentially harmful microorganism in the production process, thereby making the product unfit for consumption. Although the acidity of fermented milk makes it an unfavourable medium for the survival of most bacteria,* E. coli* O157:H7 has been described to be acid-resistant as it can steadily survive in fermented milk with a pH as low as 4.0 [21]. This underscores the nonsignificant association observed between presence of the organism and the different milk types (Tables 2 and 3). Furthermore, the Hausa/Fulani nomads in Nigeria practice the same animal husbandry and traditional livestock production methods. This could be the reason behind isolation of* E. coli* from milk samples collected from the different LGAs within the study area and the nonsignificant association between the isolates recovered (*E. coli* and STEC) and the LGAs (Tables 4 and 5).

The contamination of raw milk is believed to occur as a result of poor hygiene during milking or in the process of handling milk for transportation [22]. Similarly, contaminants in fermented milk could either be introduced before the fermentation process or at a later stage in the course of transportation. However, in this study, the detection of* E. coli* O157:H7 in both raw and fermented milk by PCR amplification of* Stx1* gene concurs with earlier reports that showed presence of the gene in* E. coli* O157:H7 recovered from cow milk and beef [23, 24].

## 4. Conclusion

The findings of this study indicate presence of* E. coli* O157:H7 in raw and fermented milk sold within Sokoto metropolis. The milk products could be contaminated in the course of milk collection, processing, storage, and/or transportation, thus presenting serious public health problems especially in children, the immunocompromised subjects, and the elderly. The findings necessitate the need for hazard analysis critical control point (HACCP) in the traditional methods of milk production in order to identify potential sources of microbial contaminants and introduce appropriate prevention and control measures. Veterinary extension services in the State should reiterate on farm hygiene practices with a view to educating the Hausa/Fulani nomads on the importance of farm hygiene and the risk associated with consumption of contaminated milk. Emphasis should be made on the zoonotic potential of* Escherichia coli* O157:H7 and the role of cattle in the spread of the pathogen. Health authorities in Sokoto State and the country at large need to regulate the production and sell of milk and milk products by introducing periodic screening and issuance of fitness certificate to farmers.

## Figures and Tables

**Table 1:** Sequences of oligonucleotides used for PCR.

Primer	Sequence (5′ to 3′)	Expected amplicon size (bp)
*Stx1F*	ATAAATCGCCATTCGTTGACTAC	180
*Stx1R*	AGAACGCCCACTGAGATCATC	

**Table 2:** Frequency of *Escherichia coli* isolates from raw and fermented milk samples.

	Number of samples positive for *Escherichia coli*	Number of samples negative for *Escherichia coli*	Total
Raw milk	15	157	160
Fermented milk	9	98	100
Total	24	236	260

*χ* ^2^ = 0.010, *p* = 0.919, OR = 1.05, (95% CI: 0.44–2.49), OR: odds ratio, and CI: confidence interval.

**Table 3:** Frequency of *Escherichia coli* O157:H7 isolates from raw and fermented milk samples.

	Number of samples positive for *Escherichia coli *O157:H7	Number of samples negative for *Escherichia coli *O157:H7	Total
Raw milk	3	157	160
Fermented milk	2	98	100
Total	5	255	260

*p* = 0.943, OR = 0.94, (95% CI: 0.154–5.704), OR:odds ratio, and CI: confidence interval.

**Table 4:** Univariable analysis of *Escherichia coli* isolates from raw milk in three Local Government Areas (LGAs).

LGA	Prevalence (%)	*p* value	OR	Total
Sokoto South	5.45 (3/55)	REF	NA	NA
Sokoto North	12.90 (8/62)	0.181	2.57	0.65–10.21
Dange-Shuni	9.30 (4/43)	0.468	1.78	0.38–8.41

OR: odds ratio, CI: confidence interval, REF: reference category, and NA: not applicable.

**Table 5:** Frequency of *Escherichia coli* O157:H7 isolates from raw milk in two Local Government Areas (LGAs).

LGA	Number of samples positive for *Escherichia coli *O157:H7	Number of samples negative for *Escherichia coli *O157:H7	Total
Sokoto North	2	6	8
Sokoto South	1	2	3
Total	3	8	11

*p* = 0.998, OR = 1.50, (95% CI: 0.084–26.86), OR: odds ratio, CI: confidence interval, and LGA: Local Government Area. Wamakko LGA was not included in the analysis as none of the samples from the area were positive for *Escherichia coli* O157:H7.
